# Understanding the association between county-level unemployment and health stratified by education and income in the southwestern United States

**DOI:** 10.1038/s41598-023-49088-z

**Published:** 2023-12-11

**Authors:** Hamnah Majeed, Shyon Baumann, Haris Majeed

**Affiliations:** 1 Department of Sociology, University of Toronto, Toronto, ON M5S 2J4 Canada; 2 Institute of Medical Science, University of Toronto, Toronto, ON M5S 1A8 Canada

**Keywords:** Risk factors, Socioeconomic scenarios

## Abstract

Past research on the relationship between unemployment rates and population health has produced mixed findings. The relationship can be influenced by the kinds of health outcomes observed, time frame, level of geographic aggregation, and other factors. Given these mixed findings, there is a need to add to our knowledge about how unemployment rates and population health are related. There is limited research that examines the association of unemployment rates with both physical and mental health, while simultaneously stratifying populations by income and education levels. Using survey-based self-reported data, this first population-based study examined the association between unemployment rates and physically and mentally unhealthy days in the southwestern United States, by county-level stratification of income (high and low) as well as education (high and low), from 2015 to 2019. After controlling for covariates, associations were modelled using negative binomial regression, with autocorrelative residuals, and were reported as rate ratios (RR). Overall, we found that a 1% rise in unemployment rates was significantly associated with an increase in physically unhealthy days [adjusted RR 1.007; 95% CI, 1.004–1.011, *P* < 0.001] and mentally unhealthy days [RR 1.006; 95% CI, 1.003–1.009, *P*  < 0.001]. Upon stratification, a significant risk was found among the high education and high income category [RR 1.035; 95% CI, 1.021–1.049, *P*  < 0.001], as well as for the high education and low income category [RR 1.026; 95% CI, 1.013–1.040, *P*  < 0.001]. A better understanding of how unemployment is associated with the health of communities with different education and income levels could help reduce the burden on society through tailored interventions and social policies not only in the United States, but also in other developed nations.

## Introduction

Self-reported adult health status of both physically and mentally unhealthy days are two domains of health-related quality of life that can provide vital information regarding the health of a representative adult sample/population^1^. Studies pertaining to developed nations have indicated numerous risk factors for physical and mental health, which span biological^2,3^, environmental^4–7^, and social realms^8,9^.

Frequent or major life changes are also believed to be risk factors for physical and mental adult health and unemployment is one such change^10^. The literature has extensively documented a positive association between individual-level adult unemployment and poor individual-level health outcomes in developed nations. It is possible that these individual-level mechanisms may scale up to serve as mechanisms at the aggregate level. Specifically, unemployed adults tend to have higher levels of chronic diseases and mental health problems, relative to those who are employed^11–16^. Potential mechanisms to explain the relationship between unemployment and poor physical and mental health include unstructured lifestyle^17,18^, feelings of marginalization and negative self-perception^18–20^, reduced income and the stress of inadequate income^21–23^, and, after the onset of unemployment, engaging in increased destructive behavior, such as smoking and excessive alcohol consumption^23^, which in turn can further harm physical and mental health^18,24,25^. Crucially, recent work using panel data supports the argument that the strongest causal influence runs from unemployment status to health conditions at the individual level^26^.

There is comparably very little work at the aggregate level. However, past work has found evidence to suggest that contextual local unemployment rates can negatively affect a population’s physical^27^ and mental^28^ health regardless of employment status^29^. Potential mechanisms at the aggregate level include restructuring of job routines, which can lead to increased stress, or a decrease in employment opportunities might result in both a deterioration of general working conditions and an increased tendency for workers to endure such conditions, such as precarious, insufficient, or unsatisfying work^30^. Unemployment may also cause deterioration in family structures and social ties, which can increase the likelihood of the subsequent formation of less healthful daily habits^31^.

Prior research has found that low socioeconomic status can lead to lower physical and mental health, due to a variety of potential reasons^32^. In the United States, low socioeconomic status groups may have restricted access to healthcare services due to coverage and cost^32,33^, receive fewer diagnostic tests and medications for chronic diseases, and feel less satisfied with the care they receive due to physicians’ perceptions that they are less likely to comply with medical advice and return for follow-up visits^32,34^. Furthermore, unemployment combined with low socioeconomic status can be even more detrimental to physical and mental health, often due to similar reasons as mentioned above. However, it is important to note that unemployment is related to low socioeconomic status, but is not the same thing as socioeconomic status, because it has different characteristics – not only does it reduce income, but it also affects self-perception (i.e., people can develop negative self-concepts when they lose their job or are not working). Of course, it does not reduce education^35^. Crucially, the association of unemployment with physical and mental health can vary by levels of socioeconomic status (i.e., education and income levels), where unemployment’s effects are stronger for low socioeconomic groups or communities^36^. To our knowledge, there is no study that has documented the association of unemployment rates with rates of physical health and mental health, while simultaneously considering differing county education and income levels as stratification parameters.

The purpose of this study is to examine the risk of unemployment for physically and mentally unhealthy days in southwestern United States, by county-level stratification of income and education from 2015 to 2019. The geographical focus was the southwest since the unemployment rate in this region has historically been relatively higher than the national rate^37^. There is also heterogeneity in income and education levels amongst counties, which makes the stratification appropriate^37^. There are two primary research questions addressed by this study: (1) What is the association of unemployment with physically unhealthy days and mentally unhealthy days (assessed separately) in southwestern United States? (2) Does the association differ when stratifying counties by income and education levels? Based on prior studies, we hypothesize that there will be a positive significant relationship between unemployment and physically and mentally unhealthy days, and such associations should vary across different education and income county-levels. This work points to the importance of socioeconomic stratification. A better understanding of how unemployment influences the health of communities with different education and income levels could help reduce its burden on individuals and society through tailored interventions and social policies.

## Results

The mean unemployment for southwestern United States during the timeframe studied was about 7.6%. The mean physically and mentally unhealthy days were 4.1 days and 3.9 days, respectively. Key summary statistics are shown in Table 1.

**Table 1:** Summary statistics of outcomes, exposures, and covariates, 2015–2019.

Variable	Minimum	Mean	Standard Deviation	Maximum
Unemployed (%)	2.68	7.63	3.28	27.65
Physically Unhealthy Day	2.6	4.06	0.61	6.5
Mentally Unhealthy Day	2.6	3.92	0.51	9.2
Social Association Rate (per 10,000)	2.17	7.76	3.99	32.82
Income Inequality	2.76	4.75	0.69	7.52
Females (%)	35.57	49.27	2.38	52.19
Rural (%)	0	35.91	29.38	100
Non-Hispanic White (%)	8.42	52.63	19.58	86.18
≥ 65 years old (%)	8.79	17.99	5.93	40.37
College Education (%)	21.87	56.73	10.53	86.60
Household Income (USD)	27,151	51,862.27	16,840.89	118,468

**Figure 1:**
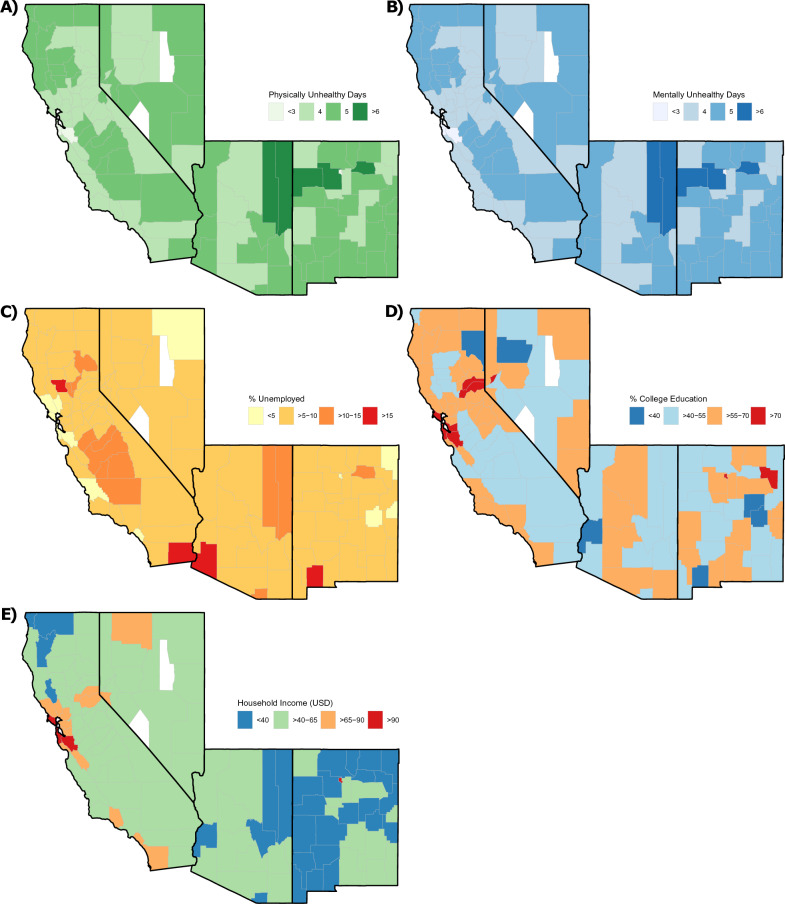
Geospatial maps depicting the mean of (**A**) physically unhealthy days, (**B**) mentally unhealthy days, (**C**) percent unemployed, (**D**) percent college education attainment, and (**E**) household income (USD), by county in southwestern United States, for the period of 2015–2019. Figure was generated using RStudio 4.0.4 (https://www.support-rstudio-com.netlify.app/products/rstudio/download/#download).

Figure 1 shows the geospatial mean of physically unhealthy days, mentally unhealthy days, percent unemployed, household income (USD), and percent college education attainment, respectively, by county in southwestern United States, for the period of 2015–2019. Generally, counties with greater physically unhealthy days have greater mentally unhealthy days and vice versa, and those counties also tend to have a higher percentage of unemployment, along with lower household income and percent college education attainment. However, these relationships are not always straightforward.

Prior to stratifying the analysis by the four education and income groups, the association between unemployment, physically and mentally unhealthy days for southwestern United States from 2015 to 2019 is shown in Table 2. A significant positive association between unemployment and physically unhealthy days [RR 1.007; 95% CI, 1.004–1.011, *P* < 0.001] and mentally unhealthy days [RR 1.006; 95% CI, 1.003–1.009, *P* < 0.001] was found. From Table 2, unemployment was found to be a significant risk factor for adult physical and mental health. We further specified these relationships by county education and income levels.

**Table 2:** Association between unemployment and physically/mentally unhealthy days in southwestern United States, controlling for covariates from 2015 to 2019.

Variable (*n* = 593)	RR (95% CI)	
	Physically unhealthy days	Mentally unhealthy days
% Unemployed	1.007 (1.004–1.011), P < 0.001	1.006 (1.003–1.009), *P* < 0.001
Social Association Rate	0.995 (0.992–0.998), P < 0.001	0.995 (0.992–0.997), *P* < 0.001
Income Inequality	1.009 (0.995–1.023), P = 0.22	1.013 (1.000–1.025), *P* = 0.045
Labor Force	1.000 (1.000–1.000), P = 0.75	1.000 (1.000–1.000), *P* = 0.73
% Females	1.010 (1.006–1.015), P < 0.001	1.013 (1.009–1.017), *P* < 0.001
% Rural	1.001 (1.000–1.001), P < 0.001	1.001 (1.001–1.002), *P* < 0.001
% Non-Hispanic White	1.000 (0.999–1.001), P = 0.49	1.001 (1.000–1.002), *P* = 0.004
% Aged 65 +	0.998 (0.996–1.000), P = 0.11	0.997 (0.996–0.999), *P* = 0.010
Year	1.016 (1.009–1.023), P < 0.001	1.027 (1.020–1.034), *P* < 0.001
State [ref: Arizona]		
California	0.961 (0.934–0.989), P = 0.007	0.971 (0.944–0.999), *P* = 0.04
Nevada	1.039 (1.001–1.078), P = 0.046	1.026 (0.989–1.064), *P* = 0.17
New Mexico	1.029 (0.995–1.065), P = 0.10	0.976 (0.945–1.009), *P* = 0.16
Education [ref: High]		
Low	1.069 (1.048–1.092), P < 0.001	1.049 (1.03–1.068), *P* < 0.001
Income [ref: High]		
Low	1.126 (1.102–1.151), P < 0.001	1.090 (1.069–1.111), *P* < 0.001

**Figure 2:**
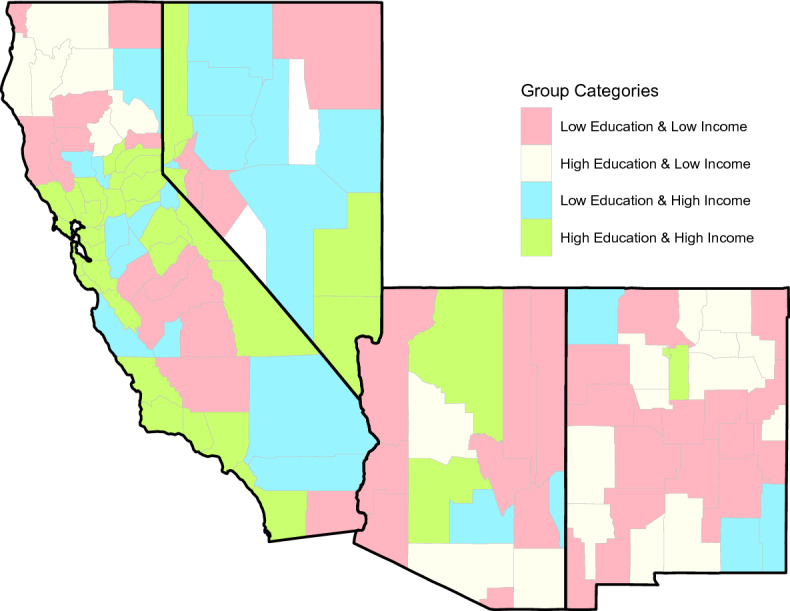
Geospatial map depicting the education and income categories (low education – low income, high education – high income, low education – high income, and high education – low income), by county in southwestern United States, for the period of 2015–2019. Figure was generated using RStudio 4.0.4 (https://www.support-rstudio-com.netlify.app/products/rstudio/download/#download).

Figure 2 geospatially illustrates the counties organized into the four categories of interest. There is substantial variation in terms of education and income groups when comparing the counties. New Mexico has relatively more low education and low income counties, whereas Nevada has relatively more low education and high income counties. California has relatively more high education and high income counties.

**Figure 3:**
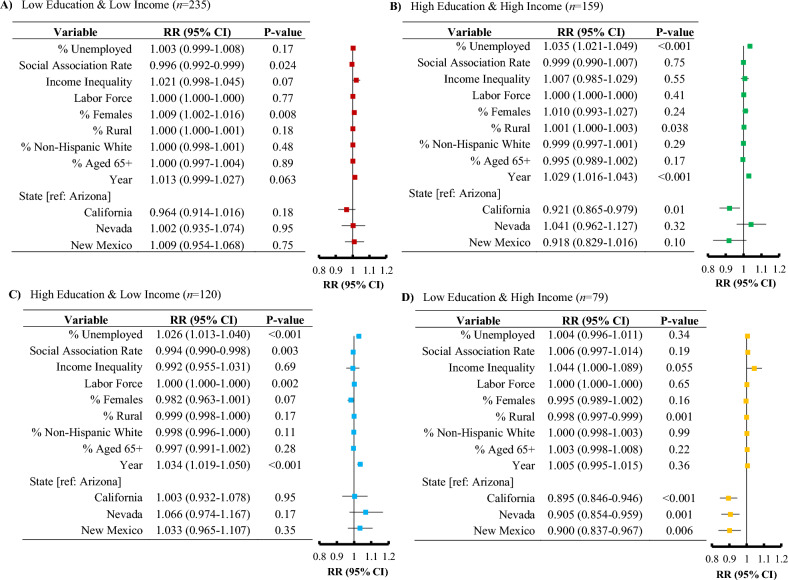
Forest plots depicting the association between physically unhealthy days and unemployment, along with various covariates in southwestern United States, for the period of 2015–2019, according to the education and income categories.

**Figure 4:**
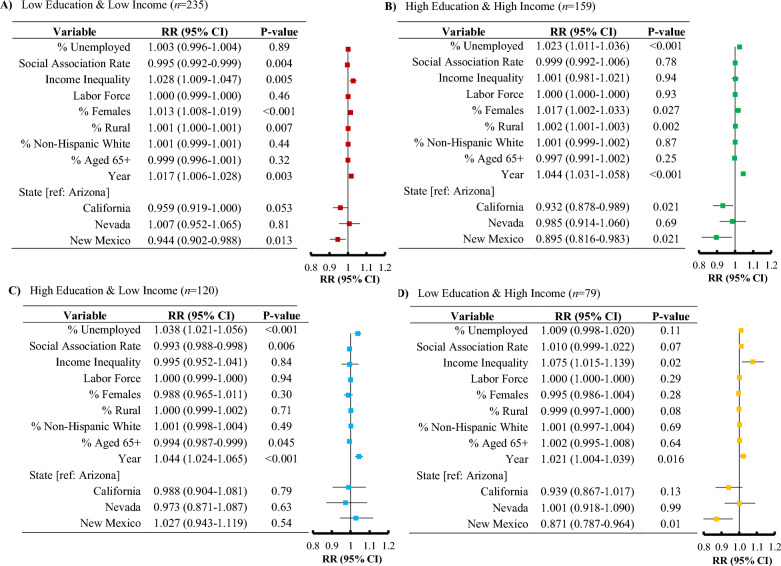
Forest plots depicting the association between mentally unhealthy days and unemployment, along with various covariates in southwestern United States, for the period of 2015–2019, according to the education and income categories.

Figures 3 and 4 depict the associations between unemployment and physically and mentally unhealthy days, respectively, stratified by county education and income levels. For the association between unemployment and physically unhealthy days (Fig. 3), unemployment resulted in a significant risk among the high education – high income group [RR 1.035; 95% CI, 1.021–1.049, *P* < 0.001], as well as for the high education – low income group [RR 1.026; 95% CI, 1.013–1.040, *P* < 0.001].

Similarly, for the association between unemployment and mentally unhealthy days (Fig. 4), unemployment resulted in a significant risk among the high education – high income group [RR 1.023; 95% CI, 1.011–1.036, *P* < 0.001], as well as for the high education – low income group [RR 1.038; 95% CI, 1.021–1.056, *P* < 0.001].

## Discussion

In this study, we found a significant positive association between unemployment and physically and mentally unhealthy days, consistent with previous studies conducted in the United States^21,38,39^. Yet to our knowledge, this is the first population-based study that assessed the association between unemployment and physically and mentally unhealthy days based on stratification of various county education and income levels in southwestern United States.

Among developed nations, previous studies have found that the risk of unemployment on overall adult health are more pronounced among those individuals belonging to a lower social class or previously earning a low wage^40^. In terms of economic drivers, unemployment, low income, and poverty are known to have detrimental impacts on adult health^41,42^. In this study, upon county-level stratification by income and education, we found a significant positive association between unemployment and physically and mentally unhealthy days in two categories: high education – high income as well as high education – low income.

We discuss here several possible hypotheses or reasons why unemployment within the high education – high income category significantly raises the risk for both physically and mentally unhealthy days. One potential reason is that regions with high education – high income tend to have better employment conditions at baseline, which include good employer health benefits. But when such regions experience more unemployment, such as during a recession, this coverage is lost. Unemployment is also associated with reduced food consumption and reduced use of preventive care services in the general population, leading to increased stress and poor physical/mental health outcomes^43^. The decline in financial stability and in social status that can come with unemployment can create a discrepancy with people’s self-perceptions and with how they imagine or desire others to perceive them. This discrepancy might have direct negative effects on emotional well-being^44^. This individual-level tendency might also scale up so that there is a discrepancy between individuals’ self-perceptions and how they imagine others view their county in negative or positive terms. To the extent that individuals identify with their county, this could affect their mental health. This discrepancy might also reduce the availability or salience of some social ties, which in turn reduces resources that people rely on for emotional well-being^10^. The harm to these social ties can result in adverse physical and mental health outcomes.

Regarding the high education – low income category, the mechanisms leading to increases in physically unhealthy and mentally unhealthy days might be slightly different, but with overlap as well. As with the high education – high income group, unemployment can be discrepant with self-perceptions and reduce social ties. Furthermore, this group experiences relatively more financial hardship, and unemployment is likely to exacerbate the relationship between low financial resources and poor health. One further mechanism to consider behind the negative relationship between unemployment and health is a social norm effect^45,46^. If people experience a hardship that is also experienced as outside social norms, rather than widely shared, the impact can be felt more acutely. Moreover, these counties might be especially vulnerable to rising unemployment rates’ deleterious effect on working conditions, which would force people to endure more stressful and less satisfying employment due to a reduction in alternatives^30^. More research is needed to better explore the reasons why these two groups were found to have a significant positive association between unemployment and physically and mentally unhealthy days.

An alternative explanation for the associations we see takes a different perspective on the relationship between unemployment and health. Compared to high-education counties, where we see significant associations with negative health outcomes, there could perhaps be a “capping off” of negative health outcomes in low-education counties because those counties generally have poor health, thereby washing out the specific relationship with unemployment.

The findings in this study are subject to several limitations. First, the data is based on surveys, which may be subject to numerous biases, such as recall bias, selection bias, and accessibility issues^47^. Regarding recall bias, some of the past research relies on more objective measures such as mortality. While our findings are consonant with that past research on the relationship between unemployment and health^48,49^, the use of more objective health measures would increase confidence in the associations we find, especially the measure of mortality, which allows for insight into suicide rates. Regarding selection effects, further research should seek to understand how selection effects might produce significant associations between unemployment and poor health within high-education counties. Second, the study design is ecological, where the unit of analysis are counties, but there is likely to be heterogeneity in education and income levels when comparing city to city, so this is a scale-related limitation^50^. Third, sex-specific outcome data was not available, yet the risk of unemployment on adult health is known to vary by sex^43,51^. Fourth, the sample size for the number of counties within each of the four categories was not identical, leading to differing statistical power for such associations based upon stratifications. Fifth, our data was based on annual measures; future work can benefit from data that allows for analysis that is temporally more fine-grained, than annual measures of unemployment and health, and also examines longer time frames so as to model longer lags between unemployment and health measures^48,52^. Sixth, causal effect cannot be established with the analysis presented in this paper. There is the possibility of reverse causality, whereby poor health can be a determinant of unemployment^53^. Lastly, there is the possibility of unobserved variation between counties that may bias the results through its impact on both unemployment and health. Despite these limitations, the number of physically and mentally unhealthy days is a valid indicator for county health status and can serve as a useful measure for monitoring the health of a local population^38^.

## Conclusion

Findings of this study are novel and suggest that unemployment has varying health associations across differing county education and income levels in the southwestern United States. Further studies are encouraged to consider socioeconomic parameters when understanding the association of unemployment with both mental and physical health, since the relationship can vary in terms of individual or population social contexts and social class categories. More work is also needed regarding the establishment of a causal relationship and the nature of causal mechanisms between unemployment and health at the aggregate level.

## Methods

### Study design

A population-based study was conducted for the pooled years of 2015–2019 using annual data obtained from the Robert Wood Johnson Foundation (https://www.countyhealthrankings.org/). The Robert Wood Johnson Foundation collaborates with the Behavioral Risk Factor Surveillance System (BRFSS) US data based on random digit dial telephone survey that is conducted annually in all states, counties, and US territories^54^. Data obtained from the BRFSS are representative of each state’s total non-institutionalized population > 18 years of age and have included more than 400,000 annual respondents with landline telephones or cellphones since 2011 through surveys and all measures are based on self-reports^54,55^.

There were two outcome variables of interest: physically and mentally unhealthy days. Physically unhealthy days is defined as the average number of physically unhealthy days reported in the past 30 days (age-adjusted). This measure is based on responses to the BRFSS question: “now thinking about your physical health, which includes physical illness and injury, for how many days during the past 30 days was your physical health not good”? Whereas mentally unhealthy days is defined as the average number of mentally unhealthy days reported in the past 30 days (age-adjusted)^55^. The responses are based on the BRFSS question: “Now thinking about your mental health, which includes stress, depression, and problems with emotions, for how many days during the past 30 days was your mental health not good”? The primary exposure variable was the percent of unemployed adults within each county, defined as the percentage of population aged > 16 years unemployed and looking for work in any sector^55^.

The region of interest was the southwestern United States, where unemployment rates have consistently remained higher than the nation’s average for several decades^37^. Our dataset included the following sample size: California (2015, *n* = 50; 2016, *n* = 58; 2017, *n* = 58; 2018, *n* = 58; 2019, *n* = 58), Nevada (2015, *n* = 14; 2016, *n* = 16; 2017, *n* = 15; 2018, *n* = 15; 2019, *n* = 15), Arizona (2015, *n* = 15; 2016, *n* = 15; 2017, *n* = 15; 2018, *n* = 15; 2019, *n* = 15), and New Mexico (2015, *n* = 29; 2016, *n* = 33; 2017, *n* = 33; 2018, *n* = 33; 2019, *n* = 33), where ‘*n*’ is the number of counties in each state for all five years. A total of 593 counties were included in the analysis.

### Data structure

Counties in the southwest were stratified by four groups, according to varying income (i.e. adult median household income in USD) and education levels (i.e. percentage of adults with some post-secondary education)^37^. Since our dataset spanned five years, the total sample size was 593. The four categories were as follows: low education – low income (*n* = 235), high education – high income (*n* = 159), low education – high income (*n* = 79), and high education – low income (*n* = 120). These categories were based on above- or below-average education and income level throughout the study period of 2015–2019. For instance, the mean percent of college educational attainment in southwestern United States was ~ 56%, hence counties with ≤ 56% were defined as ‘low’ education, and counties with > 56% were defined as ‘high’ education. Similarly, the median household income for the southwest was $51,862 (USD). Counties with ≤ $51,862 were defined as ‘low’ income and counties with > $51,862 were defined as ‘high’ income.

### Statistical analysis

The first objective of this study was to understand stratified county-level associations between; (1) unemployment and physically unhealthy days and; (2) unemployment and mentally unhealthy days in southwestern United States from 2015 to 2019 (see Supplementary Fig. 1). Various covariates included the following: social association rate (number of membership associations per 10,000 population), income inequality (ratio of household income at the 80th percentile to income at the 20th percentile), labor force (size of the labor force), percent females, percent rural, percent non-Hispanic white, percent aged ≥ 65 years old, median household income (income where half of households in a county earn more and half of households earn less), percent college education (percentage of adults ages 25–44 with some post-secondary education), as well as the states/regions and the trend (i.e. year)^56^.

From 2015 to 2019, both outcome variables had a significant rising trend, along with the presence of autocorrelation (i.e., the tendency for health conditions to be highly correlated from the previous to the subsequent year)^57^. Based on the differences in variance and mean, along with data distribution, there was a clear indication of skewness in the outcome variables. Unlike conventional ordinary least squares regression, generalized linear models do not require the response variable to be transformed every time to have a normal distribution, and so modeling is more flexible. For this reason, a generalized linear model was chosen for our analysis. In particular, negative binomial regression, with residuals of autoregressive order one (i.e. AR(1) model)^57^ was used to assess the association between physically and mentally unhealthy days and unemployment, upon controlling for the covariates mentioned above. A detailed statistical analyses of the choice of model (i.e. ordinary least squares regression versus negative binomial regression models) has been provided in Supplementary Table 1. The estimated parameters of the coefficient were interpreted as a rate ratio (RR), which corresponds to a 1-unit increase in the exposure variables. Unadjusted models have been added in Supplementary Table 2.

The second objective was to determine if the relationship differs by varying county income and education levels, thus the associations between unemployment, physically and mentally unhealthy days were stratified by the four categories mentioned above using negative binomial regression, with residuals of AR(1) model.

A sensitivity analysis using conventional ordinary least squares regression confirmed that negative binomial regression is the better technique for analyzing these data. All confidence intervals (CI) were computed at 95%, along with two-sided Student’s *t*-tests evaluated at type I error of *P* < 0.05. All computation, analyses, and figure creation used a combination of Microsoft Excel (version 2013), RStudio (version 4.0.4), and STATA (version 15).

### Supplementary Information

Supplementary Information.

## Data Availability

Data was obtained from the Robert Wood Johnson Foundation: https://www.countyhealthrankings.org/ Further data for US Census Population estimates can be found through CDC WONDER database: https://wonder.cdc.gov/population-projections.html The BRFSS data is available through: https://nccd.cdc.gov/BRFSSPrevalence/rdPage.aspx?rdReport=DPH_BRFSS.ExploreByTopic&irbLocationType=StatesAndMMSA&islClass=CLASS08&islTopic=TOPIC24&islYear=2022&rdRnd=45274.
