# Supplementary material for: Understanding the association between county-level unemployment and health stratified by education and income in the southwestern United States
Source: Sci Rep. 2023 Dec 11;13:21988. doi: 10.1038/s41598-023-49088-z (PMC10713646; doi:10.1038/s41598-023-49088-z)
Supplement: Supplementary file 1 — Supplementary Information. [file 41598_2023_49088_MOESM1_ESM.docx]

**Supplementary Online Content**

**Understanding the association between unemployment and health based on county-level education and income stratification in southwestern United States**

Hamnah Majeed^1^, Shyon Baumann^1^, Haris Majeed^2^*

*Corresponding Author: Haris Majeed (haris.majeed@utoronto.ca)

^1^ Department of Sociology, University of Toronto, Toronto, Ontario, Canada M5S 2J4

^2^ Institute of Medical Science, University of Toronto, Toronto, Ontario, Canada M5S 1A8

*Author to whom correspondence should be addressed: haris.majeed@utoronto.ca

**Supplementary Figure 1.** Scatterplots stratified by various education and income levels of physically and mentally unhealthy days versus unemployment in southwestern United States counties from 2015-2019.

**Supplementary Table 1**: Goodness of fit tests for chosen models.

| **Stratification** | **Variable** | **Model Type** | **LogLink*** | **Likelihood Ratio Test (P-value)** |
| --- | --- | --- | --- | --- |
| High Education  & Low Income | Physically Unhealthy Days | OLS | -36.4 |  |
|  |  | Negative Binomial | 63.8 | 200.4 (P<0.001) |
|  | Mentally Unhealthy Days | OLS | -87.6 |  |
|  |  | Negative Binomial | 45.1 | 265.4 (P<0.001) |
| High Education  & High Income | Physically Unhealthy Days | OLS | -24.9 |  |
|  |  | Negative Binomial | 106.2 | 262.3 (P<0.001) |
|  | Mentally Unhealthy Days | OLS | -4.1 |  |
|  |  | Negative Binomial | 122.8 | 253.8 (P<0.001) |

* Likelihood ratio test

**Supplementary Table 2**: Unadjusted associations between unemployment and health, controlled for state, 2015-2019.

| **Variable** | Outcome (RR, P-value):  **Physical Unhealthy Days** | Outcome (RR, P-value):  **Mental Unhealthy Days** |
| --- | --- | --- |
| Unemployment | 1.019 (P<0.001) | 1.011 (P<0.001) |
| State  Arizona  California  Nevada  New Mexico | reference  0.912 (P<0.001)  0.995 (P=0.83)  1.045 (P=0.031) | reference  0.936 (P=0.0004)  1.006 (P=0.79)  0.972 (P=0.15) |
